# Effect of Sodium Ascorbate and Delayed Bonding on the Bond Strength of Silorane and Two-step Self-etch Adhesive Systems in Bleached Enamel

**DOI:** 10.5681/joddd.2014.038

**Published:** 2014-12-03

**Authors:** Mehdi Abed Kahnemooyi, Amir Ahmad Ajami, Soodabeh Kimyai, Fatemeh Pournaghiazar, Siavash Savadi Oskoee, Mohammad Ali Mhammadi Torkani

**Affiliations:** ^1^Dental and Periodontal Research Center, Tabriz University of Medical Sciences, Tabriz, Iran; ^2^Associate Professor, Department of Operative Dentistry, Faculty of Dentistry, Tabriz University of Medical Sciences, Tabriz, Iran; ^3^Assisstant Professor, Department of Operative Dentistry, Faculty of Dentistry, Tabriz University of Medical Sciences, Tabriz, Iran; ^4^Professor, Department of Operative Dentistry, Faculty of Dentistry, Tabriz University of Medical Sciences, Tabriz, Iran; ^5^Postgraduate Student, Department of Operative Dentistry, Faculty of Dentistry, Tabriz University of Medical Sciences, Tabriz, Iran

**Keywords:** Delayed bonding, enamel bleaching, shear bond strength, silorane-based adhesives, sodium ascorbate

## Abstract

***Background and aims.*** Studies have shown decreased bond strength of composite resin to human and bovine bleached enamel. This study evaluated the effect of sodium ascorbate and delayed bonding on the bond strength of two adhesive systems to bleached enamel.

***Materials and methods.*** The labial surfaces of 150 sound bovine incisor teeth were abraded with abrasive paper. The teeth were randomly divided into 8 groups: A: control; B: bleached with 35% hydrogen peroxide; C: bleached with 35% hydrogen peroxide + sodium ascorbate gel; and D: bleached with 35% hydrogen peroxide + delayed bonding. In groups A‒D, silorane adhesive system and Filtek silorane composite resin were used. In groups E‒H, the same preparation methods of groups A-D were used. Two-step self-etch Clearfil SE Bond adhesive systems and AP-X composite resin were administered. Shear bond strength of each group was measured. Two samples were prepared for each surface preparation for ultra-structural evaluation. Two-way ANOVA and Tukey test were used for data analysis at P<0.05.

***Results.*** The interaction between the adhesive system type and surface preparation protocol was significant (P=0.014), withsignificant differences in shear bond strengths in terms of the adhesive systems (P<0.01). There were significant differences in shear bond strength in terms of surface preparation techniques irrespective of the adhesive system (P<0.01).

***Conclusion.*** The results showed that bleaching with 35% hydrogen peroxide decreased the shear bond strength values with both adhesive systems, and a one-week delay in bonding and 10% sodium ascorbate for10 minutes restored the bond strength in both adhesive systems.

## Introduction

Beaching of vital teeth with carbamide peroxide or hydrogen peroxide has been established as a safe and conservative treatment modality.^1,2^ Ever-increasing popularity of tooth bleaching as a conservative and esthetic treatment makes knowledge about the effect of bleaching agents on adhesive restorations and bonding agents important.^3^ Several studies have shown a decrease in bond strength of composite resin to human and bovine bleached enamel.^4,5^ A decrease in bond strength immediately after bleaching of teeth is a concern and is of great clinical significance. In most cases, tooth bleaching is carried out before esthetic restorative procedures.^4^ The decrease in bond strength has been attributed to the oxygen released from bleaching agents into tooth structure,^6,7^ changes in the mechanical properties of enamel and dentin and denaturing and instability of proteins forming the fibril structure of the organic matrix of dentin.^7,9^

In addition, bonding process immediately after tooth bleaching affects the marginal seal of composite resin restorations, resulting in microleakage,^9^ which can lead to marginal discoloration, recurrent caries and pulp inflammation after composite resin restorations.^10^ It has been reported that a delay in bonding procedure from 24 hours to 2 weeks after the bleaching procedure restores the bond strength.^11,6,12-14^, In addition, it has been claimed that use of ascorbic acid,^13^ catalase,^14^ ethanol^15^ and water^16^decrease peroxides and other oxygen free radicals. It has been reported that application of 35% sodium ascorbate for 2 minutes eliminates oxygen residues comparable to a 5-day delay in bonding.^17^Tooth bleaching compromises formation of resin tags.^18^The oxygen pooled in tooth structure prevents polymerization of monomers in the adhesive systems.^19^

Although the bond strength decreases after tooth bleaching, the inhibitory effect of oxygen in various bonding systems is different due to the water content of their hydrophilic monomers.^20^Siloxanes present in silorane composite resins are responsible for a high rate of hydrophobicity of these composite resins. The bonding agent of the silorane adhesive systems has hydrophobic dimethacrylate monomers (70-80 wt%) for its compatibility with silorane composite resin. In addition, the bonding agent of this system is devoid of HEMA, which is a hydrophilic monomer, although the adhesive system of Clearfil SE Bond contains hydrophilic HEMA, 10-MDP (10-methacryloyloxy decyldihydrogen phosphate) and aliphatic dimethacrylates.^21^ Therefore, it might be expected that the silorane adhesive system will react differently to tooth bleaching procedures.

On the other hand, sodium ascorbate exerts different effects on the bond strength of different adhesive systems to bleached dentin^22^ and bond strength recovery to the level of the un-bleached enamel takes place at different times.^23^ Concerns about the effect of bleaching agents on the bond strength raises the question whether new bonding systems can overcome the undesirable effects of bleaching agents or not.

The aim of the present study was to evaluate the effect of sodium ascorbate and delayed bonding on the bond strength of silorane adhesive system and a two-step self-etch bonding system to bleached enamel.

## Materials and Methods

In this in vitro study, 150 sound bovine incisor teeth were used. The teeth were stored in 0.1% thymol solution until all the samples were collected.^31^Teeth with no cracks or enamel defects were selected for the study. Soft tissue remnants were removed from root surfaces. The teeth were vertically mounted in auto-polymerizing acrylic molds (Acropons, Tehran, Iran) with tooth crowns out of the acrylic resin. All the labial tooth surfaces were abraded with 240-grit silicon carbide paper to achieve a smooth surface; 600-grit silicon carbide paper was used for the final polish and to achieve a standard smear layer. The teeth were randomly divided into 8 groups (n=19) from A to H.

### Group A

The teeth were stored in artificial saliva at 37°C for a week.^24^Artificial saliva was prepared from 1.5 mmol/L of sodium bicarbonate, 4.8 mmol/L of sodium chloride, 137 mmol/L of potassium chloride, 4 mmol/L of potassium dihydrogen phosphate and 100 mL of deionized water. All the tooth surfaces were covered with celluloid tape except for a round area in the center of the labial surface, measuring 2 mm in diameter. Silorane adhesive (3M ESPE, Dental Products, St. Paul, MM, USA) was applied on the relevant area according to manufacturer’s instructions. Then, cylindrical plastic molds, 2 mm in diameter and 4 mm in height, were used to place Filtek silorane composite resin (3M ESPE, Dental Products, St. Paul, MM, USA) on the enamel surface; 2-mm increments of composite resin were light-cured using Astralis 7 light-curing unit (Ivoclar, Vivadent, Liechtenstein) at a light intensity of 600 mW/cm^2^ and a tube diameter of 8 mm.

### Group B

Endo Opalescent 35% hydrogen peroxide gel (Ultradent. Products Inc., USA) was applied on the tooth surface 3 times a day for 15 minutes each time,^25^ which continued for 1 week,^32^ during which the teeth were kept in artificial saliva.^31^After one week, the tooth surface was covered with celluloid tape and silorane adhesive and Filtek silorane composite resin were added. Finally, the teeth were kept in artificial saliva at 37°C for 24 hours.^24^

### Group C

The teeth were bleached for 1 week using the same protocol as described for group B. Then 10% sodium ascorbate hydrogel was applied on tooth surfaces for 10 minutes and rinsed with distilled water for 30 seconds.^26,27^It should be pointed out that sodium ascorbate hydrogel was formulated based on a previous study.^28^

### Group D

The teeth were bleached in the same manner as those in group B. Then the teeth were stored in fresh artificial saliva for one week. Then the adhesive system and composite resin were applied.

Shear bond strength of the samples were measured in Newton using a Hounsfield test equipment (Model H5K-S, Salford, Redhill, Surrey, England) at a crosshead speed of 1 mm/min. Then the values in Newton were divided by the cross-section surface areas of the samples to achieve bond strength values in MPa. After specimen failures, the failure modes were categorized under a stereomicroscope (Nikon, Tokyo, Japan) as follows:^29^

Adhesive failure: failure at composite resin-enamel interface

Cohesive failure: failure within the enamel or composite resin

Mixed failure: a combination of the two above

In order to evaluate the surface ultrastructure of teeth 2 extra samples were prepared from tooth surfaces before application of the adhesive system and composite resin for electron microscopy (Tescan Vega II X MN, Brno, Czech Republic): control; bleached; bleached + ascorbate sodium; bleached + delayed bonding. In the control group 2 extra samples were evaluated under the electron microscope after application of the adhesive systems and rinsing with acetone.

The procedures in groups E to H were the same as those in groups A to D, respectively, and then AP-X composite resin and Clearfil SE Bond (Kuraray Co. LTD, Osaka, Japan) were used instead of Filtek silorane composite resin and silorane adhesive.

Two-way ANOVA was used to compare shear bond strength values between the groups. A post hoc Tukey test was used for two-by-two comparison of the groups. SPSS 15 was used for statistical analyses at a significance level of P<0.05.

## Results

**Graph 1.**
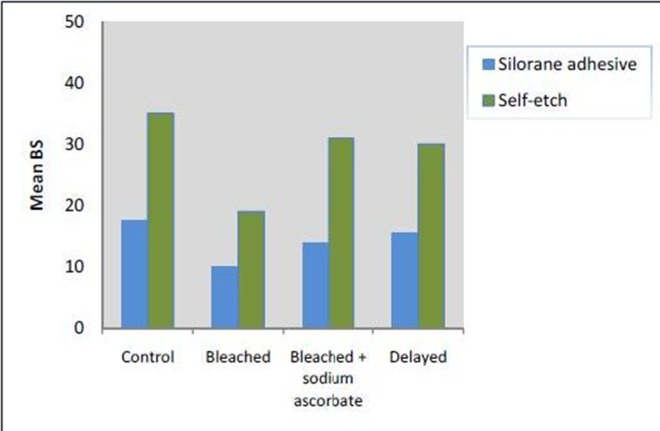
Graph 1. Error bars of the means of shear bond strength values in the groups under study in terms of surface preparation protocol.

Table 1 presents descriptive statistics of shear bond strength means in the study groups. Graph 1 presents the error bars of shear bond strength values in the study groups in terms of surface preparation protocol.

**Table 1:** Means and standard deviations of shear bond strength values in the groups under study

Adhesive system	Surface preparation technique	Mean	SD	No.
Silorane Adhesive	control	16.23^a^	5.11	19
	bleached	10.21^b^	3.22	19
	bleached + sodium ascorbate	13.37^a^	5.01	19
	bleached + delayed bonding	14.97^a^	3.93	19
Clearfil SE Bond	control	35.3^c^	10.31	19
	bleached	18.02^d^	9.48	19
	bleached + sodium ascorbate	31.23^c^	11.59	19
	bleached + delayed bonding	30.42^c^	9.13	19

Two-way ANOVA showed that the interaction between the two adhesive systems and the surface preparation protocols was significant (P=0.014, F_3,144_=3.672).

There were significant differences in the means of shear bond strength values in terms of the bonding system (P<0.01, F_1,144)_=139.86). In addition, there were significant differences in mean shear bond strength values of surface preparation protocols, irrespective of the adhesive system used (P<0.01, F_(3,144)_=14.773). Two-by-two comparisons of the mean bond strength values in groups using post hoc Tukey tests showed significant differences in the mean bond strength values between the control and bleached groups (P<0.001), and between the bleached and bleached + sodium ascorbate groups (P<0.001). However, there were no significant differences in bond strength between the other groups (P>0.05). In addition, the interaction between the two adhesive systems and the surface preparation protocols was significant (P=0.014, F_(3,144)_=3.672).

Table 2 shows the failure modes at enamel-composite resin interface for the two adhesive systems in the groups under study.

**Table 2:** Failure modes at enamel-composite resin interface for the adhesive systems in the groups under study

		Failure mode		
Adhesive system	Surface preparation technique	Adhesive	Cohesive	Mixed
Silorane Adhesive	control	19	0	0
	bleached	19	0	0
	bleached + sodium ascorbate	19	0	0
	bleached + delayed bonding	19	0	0
Clearfil SE Bond	control	6	0	13
	bleached	17	0	2
	bleached + sodium ascorbate	7	0	12
	bleached + delayed bonding	6	0	13

### SEM Findings

**Figure 1.**
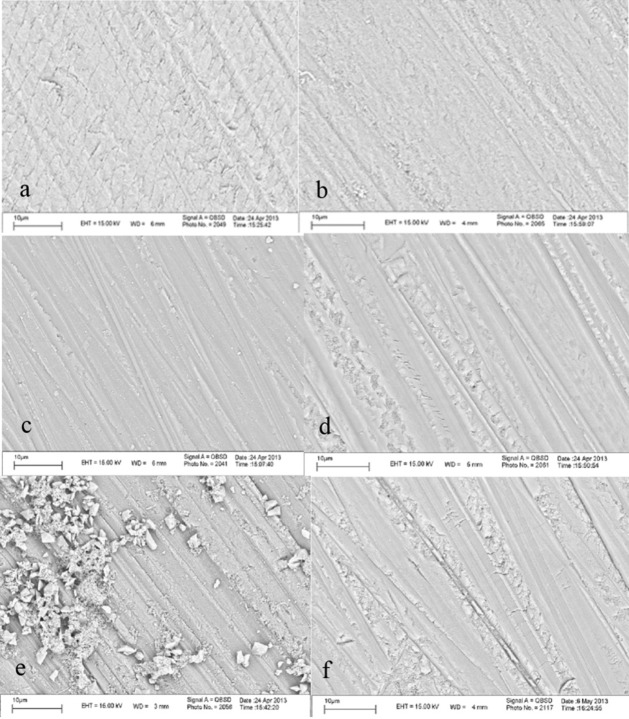
SEM observations. (a) Enamel surface after application of Clearfil SE Bond and washing with acetone. (b) Enamel surface after application of silorane adhesive and washing with acetone. (c) Enamel surface treated with 600-grit SiC paper. (d) Enamel surface treated with bleaching agent. (e) Enamel surface treated with bleaching agent and sodium ascorbate. (f) Enamel surface one week after bleaching.

Figures 1aand1bshow SEM images of the effect of two adhesive systems on enamel abraded with 600-grit abrasive paper and Figures 1c to 1f show SEM images of various preparation techniques of enamel abraded with 600-grit abrasive paper at ×5000.

Based on Figure 1a, Clearfil SE Bond adhesive system has completely eliminated the smear layer and the keyhole appearance of enamel rods is visible; this system has created some surface roughness. However, the silorane adhesive system has produced some surface roughness, which is barely visible (Figure 1b).

Figure 1d shows the effect of bleaching on abraded enamel after 5 hours and 15 minutes of bleaching during a period of 1 week. As the image shows 35% hydrogen peroxide has created local porosities which are more evident in the ridges between the grooves resulting from 600-grit abrasive paper. Figure 1f shows the same porosities one week after completion of the bleaching procedure.

Figure 1e shows the surface prepared with sodium ascorbate after bleaching of enamel, in which sodium ascorbate crystals are visible on the enamel surface.

## Discussion

The aim of the present study was to evaluate the effect of sodium ascorbate and delayed bonding on the bond strength of silorane adhesive and two-step self-etch adhesive systems to bleached bovine enamel.

In various studies, bovine teeth have been used to compare the bond strength of bleached enamel with that of unbleached enamel.^16,30^ The results of studies carried out on human teeth generally confirm the results of studies carried out on bovine teeth.^31^ In addition, a study by Nakamichi in 1983 showed that bovine teeth can properly replace human teeth to study the bond strength.^32^Based on the results of the present study, the means of shear bond strength values with both adhesive systems in all the study groups (control, bleached, bleached + sodium ascorbate and bleached + delayed bonding) were significantly different and Clearfil SE Bond exhibited a higher bond strength compared to silorane adhesive. Conversion or polymerization rate is one of the criteria for proper mechanical properties of restorative materials.^33^The rate of polymerization of the resin layer, which is produced by the adhesive systems, is a factor affecting the bond strength and its longevity.^34^The polymerization rate of Clearfil SE Bond adhesive system in the adhesive layer is higher than that of silorane adhesive system.^21^Silorane adhesive system with a pH value of 2.7 is classified as an ultra-mild self-etch adhesive compared to Cleasrfil SE Bond with a pH value of 1.8. Since the conditioner etching pattern depends, to a great extent, on the acidity of the conditioner^35^ and the main factor for bonding with enamel is penetration of resin into the microporosities on the enamel surface.^36^ It is rational to believe that the depth and pattern of etching can affect the enamel bond strength. SEM images of the present study showed that silorane adhesive cannot dissolve the smear layer whereas Cleafil SE Bond can dissolve the smear layer and can also produce enamel surface roughness. On the other hand, the type of the acidic monomers used in the chemical structure of an adhesive system can influence the bond strength by establishing a chemical bond along with micromechanical retention.^37^

10-MDP which is one of the constituents of Clearfil SE Bond can establish a stable bond with the calcium of hydroxyapatite and form stable salts on the surface.^37^The three above-mentioned mechanisms can explain the high bond strength of Clearfil SE Bond adhesive system.

In the present study, bleaching of enamel with 35% hydrogen peroxide for one week resulted in a significant decrease in shear bond strength with both adhesive systems compared to the control group. However, Akin et al^38^ and Patuscoa et al^39^ showed that the shear bond strength of brackets bonded to the enamel bleached with 10% carbamide peroxide is not affected. These results might be attributed to a lower concentration of hydrogen peroxide in 10% carbamide peroxide, which is one-tenth of its concentration in 35% hydrogen peroxide.

A decrease in bond strength subsequent to bleaching might be attributed to the presence of oxygen in enamel. It has been reported that oxygen is an inhibitory factor for polymerization reactions of free radicals. Oxygen reacts with the free radicals and oxidizes them and has a low affinity to react with monomers.^40^However, Perdigao et al reported that a decrease in bond strength after bleaching might not be attributed to the presence of oxygen in enamel; rather, dentin might function as a reservoir and pooling of oxygen in dentin might be a factor for a decrease in bond strength.^41^

Titly et al reported that the resin‒bleached enamel interface was completely different from resin‒unbleached enamel interface,^30^ i.e. in the bleached enamel large areas of enamel surface were devoid of resin and areas with resin tags were not very distinct and were not deep.^30^In addition, in another study by Titly, SEM evaluations showed that the resin–bleached enamel interface was granular and porous.^4^In the present study, the decrease in shear bond strength after bleaching was lower with silorane adhesive compared to that with Clearfil SE Bond. Cadonaro et al^16^ showed that systems with higher hydrophilic monomer content are more sensitive to bleaching procedures.On the other hand, in adhesive systems which contain hydrophilic compound in chemical structure there is a thicker oxygen inhibition zone.^27^A lower bond strength decrease in silorane adhesive might be attributed to its hydrophobic monomer content.

In the present study, there were significant differences in bond strength between groups treated with sodium ascorbate and bleached groups in both adhesive systems, with higher bond strength in groups treated with sodium ascorbate. There were no significant differences in bond strength between groups treated with sodium ascorbate and control groups with both adhesive systems, indicating that sodium ascorbate has the potential to neutralize and restore the oxidative effect of bleaching agents. The results of the present study are consistent with those of a study by Turkun et al.^42^Ascorbic acid and its sodium salt are antioxidative agents and can eliminate free radicals from the biologic environments.^43^

Lai et al^44^ showed that use of 10% sodium ascorbate can restore the lost bond strength of bleached enamel.Zhao et al^45^ showed that under certain conditions peroxide ions are exchanged with hydroxyl radicals in the apatite plexus and produce apatite peroxide, resulting in structural aberrations in the apatite plexus. It is believed that these structural aberrations can influence the biologic efficacy of biologic agents.

Lai et al^44^ proposed that use of an antioxidant can remove peroxide from the apatite plexus. Therefore, if sodium ascorbate can eliminate peroxide ions from the apatite plexus, it can reverse the effect of structural aberrations of peroxide.

In the present study, there were no significant differences between groups with delayed bonding and the control groups with both bonding systems, consistent with the results of previous studies,^12,4^indicating that a one-week delay in bonding of bleached enamel can restore the decreased shear bond strength, which is attributed to the removal of oxygen remnants from tooth structures.

SEM evaluations of groups treated with sodium ascorbate showed a large number of ascorbate crystals on enamel surfaces. Sodium ascorbate is oxidated to dihydroxyascorbate in two phases after contact with nascent oxygen. Electrochemical and topographic studies have shown that sodium ascorbate is adsorbed to smooth polymer and hydroxyapatite surfaces, which might be attributed to the strong sedimentation effect of dihydroxyascorbate, resulting from the contact of sodium ascorbate with nascent oxygen.^46^Oskuee et al^46^attributed an increase in enamel microhardness after treatment with sodium ascorbate to the presence of ascorbate crystals on the enamel surface.

All the failures at enamel-composite resin interface in all the silorane adhesive samples in all the study groups were of the adhesive type, which might be attributed to the low bond strength of this adhesive system compared to the Clearfil SE Bond system on the enamel surface. However, with the Clearfil SE Bond adhesive system in the bleached groups the majority of failures were of the adhesive type, due to lower bond strength, whereas in other groups the majority of failures were of the mixed type. Since the oral cavity environment is different from the laboratory conditions, presence of normal saliva, occlusal forces, oral moisture and temperature, and presence of microbial plaque are considered factors which can influence the results of the study. Therefore, it is suggested that the present study be carried out under clinical conditions and both adhesive systems undergo delayed bonding at different intervals so that the minimum delay in bonding can be determined for the two bonding systems. In addition, it is suggested that different concentrations and application times of sodium ascorbate be used on bleached enamel so that the effect of sodium ascorbate on bond strength of the two adhesive systems would be further evaluated.

## Conclusion

The results of the present study showed that both Clearfil SE Bond and silorane adhesive systems were affected by bleaching with 35% hydrogen peroxide and the bond strength decreased significantly. A one-week delay in bonding and use of 10% sodium ascorbate for 10 minutes restored the bond strength in both systems.
